# Diameter Tuning of $\beta$-Ga_2_O_3_ Nanowires Using Chemical Vapor Deposition Technique

**DOI:** 10.1186/s11671-017-1915-1

**Published:** 2017-03-09

**Authors:** Mukesh Kumar, Vikram Kumar, R. Singh

**Affiliations:** 1 Department of Physics, Indian Institute of Technology Delhi, Hauz Khas, New Delhi, 110016 India; 2 Nanoscale Research Facilities, Indian Institute of Technology Delhi, Hauz Khas, New Delhi, 110016 India; 3 Centre for Applied Research in Electronics, Indian Institute of Technology Delhi, Hauz Khas, New Delhi, 110016 India

**Keywords:** Gallium oxide, Nanowire, Diameter, Band-gap, Chemical vapor deposition

## Abstract

Diameter tuning of $\beta$-Ga_2_O_3_ nanowires using chemical vapor deposition technique have been investigated under various experimental conditions. Diameter of root grown $\beta$-Ga_2_O_3_ nanowires having monoclinic crystal structure is tuned by varying separation distance between metal source and substrate. Effect of gas flow rate and mixer ratio on the morphology and diameter of nanowires has been studied. Nanowire diameter depends on growth temperature, and it is independent of catalyst nanoparticle size at higher growth temperature (850–900 °C) as compared to lower growth temperature (800 °C). These nanowires show changes in structural strain value with change in diameter. Band-gap of nanowires increases with decrease in the diameter.

## Background

In the past decade, wide band-gap semiconductor nanowires have received extensive research interest due to their potential device applications [1–10]. Recently, beta gallium oxide (β-Ga_2_O_3_) with its one dimensional morphology is emerging as one of the potential semiconductor oxide nanomaterial. It has shown promising device applications including high-temperature gas sensors, UV photodetectors, high power field effect transistors (FET), and photonic switches [2, 3, 9–15]. β-Ga_2_O_3_ exhibits advantageous properties including large band-gap with E_g_ ~ 4.7–4.9 eV at room temperature (RT), high breakdown field of 8 MVcm^−1^, and outstanding thermal and chemical stability at high temperatures [11, 16–19]. It has stable β-phase, which exhibits monoclinic crystal structure [19].

Several applications of $\beta$-Ga_2_O_3_ nanostructures have been explored [3, 9, 10]. To grow, thermal chemical vapor deposition (CVD) is the most accepted and widely used technique [14, 20, 21]. It is attractive due to high deposition rate, capability of producing highly dense and pure materials, reproducibility of synthesis, and ability to control the morphology of nanostructures by controlling process parameters. For nanowire growth, vapor-liquid-solid (VLS) or vapor-solid (VS) process are well established growth mechanisms [22]. The β-Ga_2_O_3_ nanostructures including nanowires, nanosheets, nanobelts, and nanorods grown by CVD have been shown in number of reports [12, 20, 23, 24]. Nanowires as building blocks of nanodevices allow tuning of fundamental optical and electronic properties of devices by tuning nanowire diameter. Further, high aspect-ratio nanowires with different diameters are advantageous for catalytic functionalities and sensors due to large surface to volume ratio. Recently, Reddy et al. [25] have shown high photocatalytic activity of β-Ga_2_O_3_ nanorods under UV irradiation. Kumar et al. [26] have shown the catalytic activity of thermosensitive Ga_2_O_3_ nanorods. Li et al. [27] have demonstrated high performance of bridged Ga_2_O_3_ nanowires for solar-blind photodetection. Ling et al. [28] demonstrate pH sensor based on Ga_2_O_3_ nanowires and suggested possibilities for improvement in performance of pH sensors by nanowire dimensions related sensing capabilities. Conduction properties of nanowire depend on its diameter. Nanowires with diameter smaller than the depletion layer width existing due to surface state charges are completely depleted whereas nanowires with larger diameter have a conducting channel [29]. Wie et al. [30] have studied diameter-dependent band-gap alteration in strained ZnO nanowires. Therefore, diameter tuning of Ga_2_O_3_ nanowire using experimental conditions is highly desirable to tune the nanodevice properties. In this work, diameter tuning of $\beta$-Ga_2_O_3_ nanowires by CVD technique using various experimental conditions have been investigated. The dependence of nanowire diameter on separation distances between metal source and substrate has been studied. Various growth temperatures with different Au nanoparticles were explored to tune the diameter of nanowires. The nanowires with different diameters have been further investigated using XRD, Raman, and UV-vis techniques.

## Method

$\beta$-Ga_2_O_3_ nanowires have been grown on Au nanoparticles coated sapphire substrate using CVD technique where Au nanoparticles serve as catalyst. The colloidal solutions of Au nanoparticles of sizes 50 nm (with concentrations ~3.4 × 10^10^ particles/mL) and 20 nm (with concentrations ~6.8 × 10^11^ particles/mL) were purchased from Ted Pella Inc. Au nanoparticles from colloidal solution were dispersed on sapphire substrate using two-step spin-coating method (3000 rpm for 60 s followed by 9000 rpm for 30 s) and then annealed at 200 °C in the presence of argon flow (50 mL/min). Gallium metal (purity 99.999% from Sigma Aldrich) and substrate are kept in same temperature zone. Oxygen and argon gases were used to grow $\beta$-Ga_2_O_3_ nanowires. Nanowire growth has been studied systematically using various growth temperatures, different Ar/O_2_ total flow rates, different flow rate ratios, and different separation distances between source metal and substrate under pressure of 2.5 Torr.

Field-emission scanning electron microscopy (FESEM) images were recorded at RT with electron beam energy of 5 and 10 keV using Raith e-line plus system. X-ray diffraction (XRD) measurements were performed using Rigaku having CuK_α_ radiation. Raman analyses were performed at RT in backscattering configuration using an excitation wavelength of 514 nm using Horiba-LabRAM HR Evolution instrument. UV-vis spectrophotometer from Perkin Elmer (Model Lambda 1050) has been used for reflectance measurements.

## Results and Discussion

**Fig. 1:**
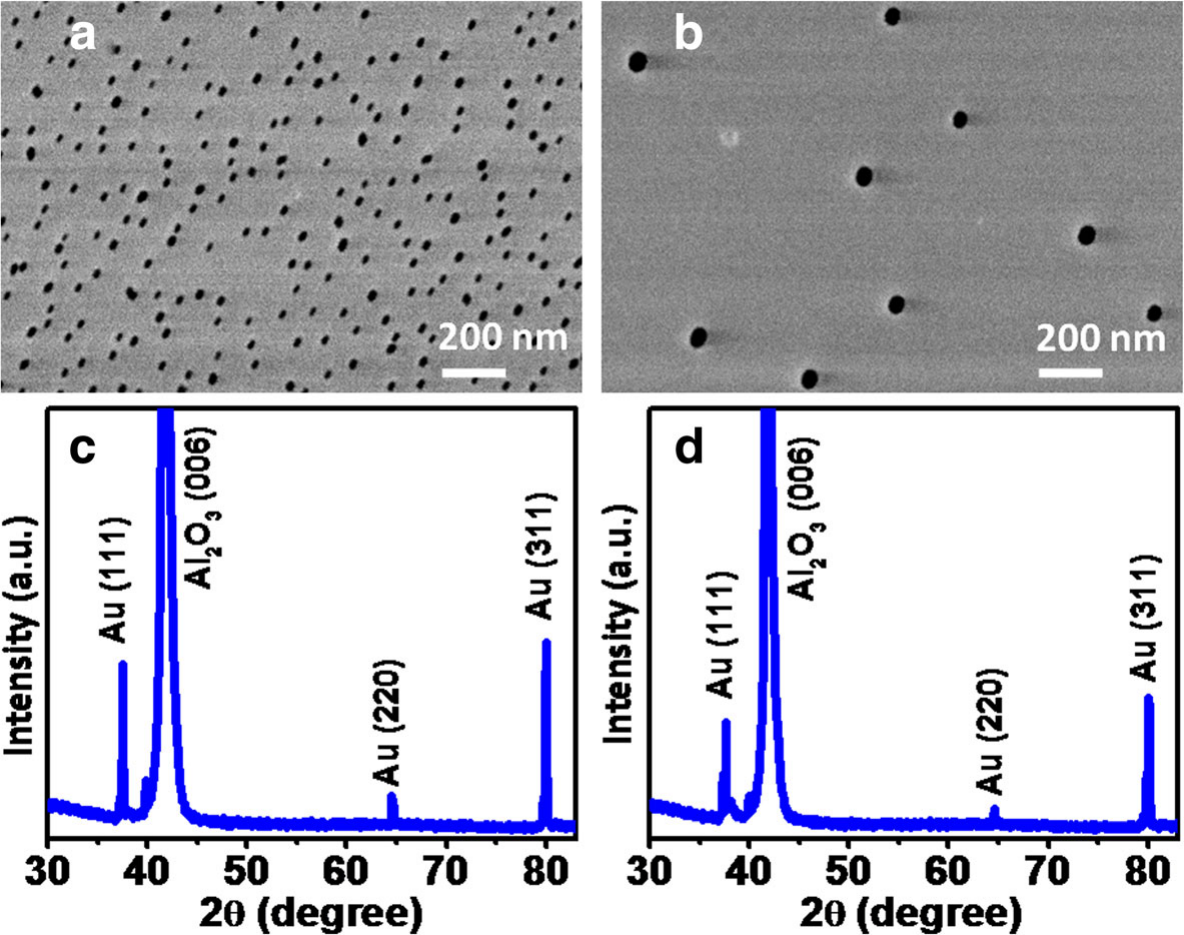
FESEM images (**a**, **b**) and XRD patterns (**c**, **d**) of dispersed Au nanoparticles with size categories of 20 and 50 nm. FESEM images demonstrate the well dispersed unconjugated Au nanoparticles. XRD confirms the presence of Au nanoparticles on sapphire substrate

Au nanoparticles (designated as 20 and 50 nm) from colloidal solutions (purchased from Ted Pella Inc.) were dispersed on sapphire substrate using spin-coating method. FESEM images and XRD patterns of dispersed Au nanoparticles with size categories of 20 and 50 nm are shown in Fig. 1a, b and c, d, respectively. A combination of low spin speed and high spin speed were used. Low spin speed spreads the colloid droplets containing Au nanoparticles. High spin speed provides the adequate outward centrifugal force to overcome the inter-particle interactions to avoid nanoparticle conjugation. FESEM images (Fig. 1a, b) demonstrate the well dispersed unconjugated Au nanoparticles. XRD patterns (Fig. 1c, d) of dispersed Au nanoparticles exhibit diffraction peaks of a typical cubic crystal structure and marked with Miller indices (hkl) according to JCPDS (Joint Committee on Powder Diffraction Standards). XRD confirms the presence of Au nanoparticles on sapphire substrate.

**Fig. 2:**
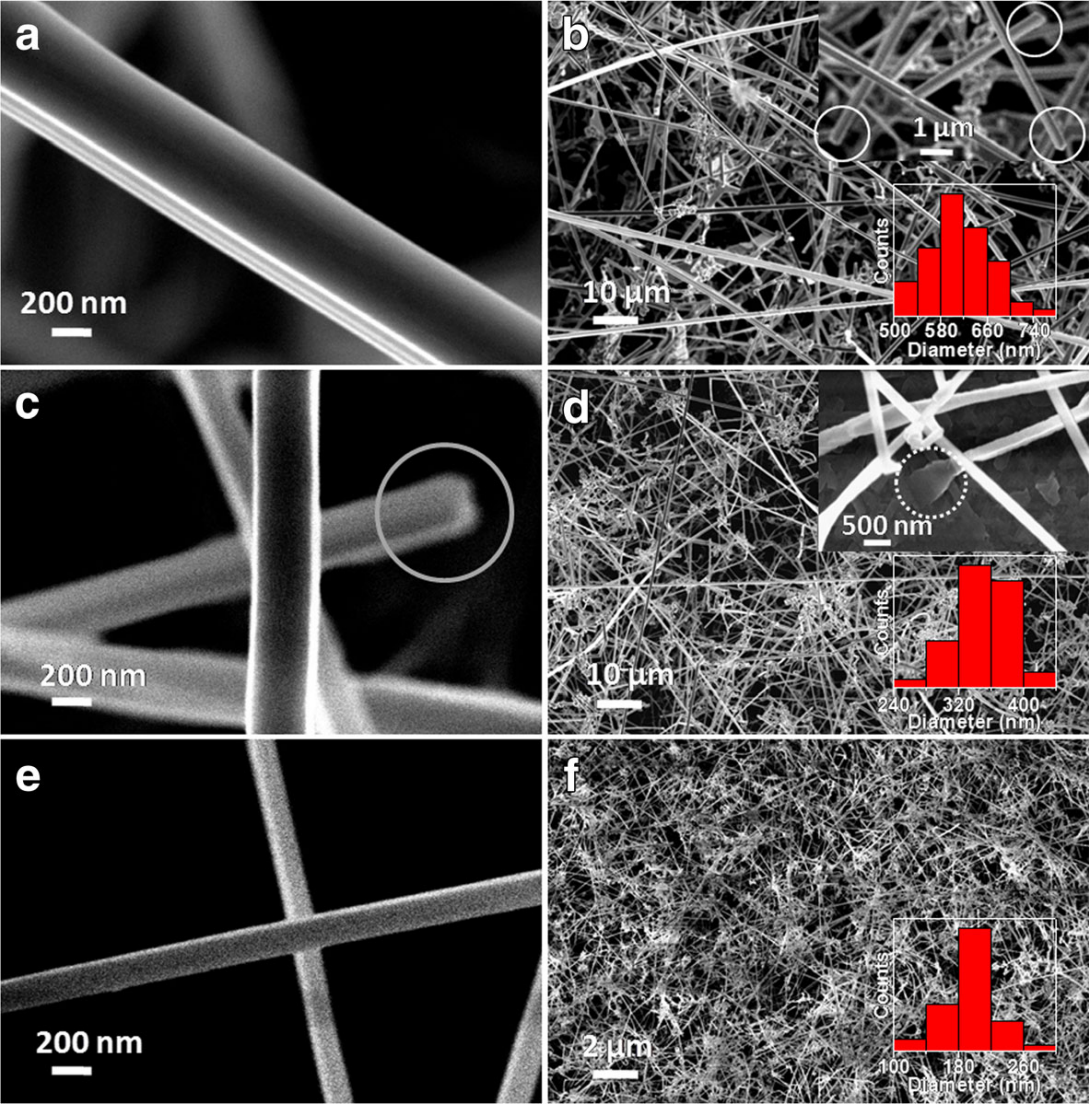
FESEM images of Ga_2_O_3_ nanowires grown at source to substrate distances of **a**, **b** 2 cm, **c**, **d** 4 cm, and **e**, **f** 6 cm where nanowire diameter decrease with increase in the separation distance on the basis of boundary layer thickness and reactant vapor species in CVD process. Circles in insets of image (**b**) and (**d**) show the tip and root of nanowire indicating root growth of Ga_2_O_3_ nanowires

**Fig. 3:**
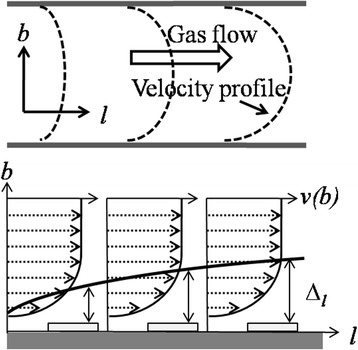
Schematic illustration of gas velocity profile v(b) and corresponding developed boundary layer (∆_*l*_) in the tube of CVD system (Reference: [23]). It shows the deposition of reactant species on wafer surface by diffusion through various thicknesses of ∆_*l*_

Ga_2_O_3_ nanowires grown at 900 °C for 30 min using 50 nm Au nanoparticles as catalyst with total gas flow rates of 80 mL/min (Ar/O_2_:3/1) have been studied using different separation distances (*S*_*d*_) between metal source and substrate (Fig. 2). Long nanowires having lengths up to several hundred of micrometers have been observed. Nanowires grown with *S*_*d*_ value of 2 cm (Fig. 2a, b) have diameter distribution mainly in range of ~500–700 nm as indicated by histogram (lower inset of Fig. 2b). These nanowires are named as D1. With increase in *S*_*d*_ value to 4 cm (Fig. 2c, d), nanowire diameter was reduced to the range of ~280–400 nm (lower inset of Fig. 2d). These nanowires are named as D2. With increase in S_d_ value to 6 cm (Fig. 2e, f), nanowire diameter was further reduced to the range of ~140–260 nm (lower inset of Fig. 2f). These nanowires are named as D3. In thermal CVD technique, deposition process for laminar gas flow at high temperature is mainly limited by mass transport [31–33]. At constant temperature, the equation governing mass transport [31, 32] is

1 $$ v(b)\frac{\partial C\ \left( l,\  b\right)}{\partial l}= D\frac{\ {\partial}^2 C\ \left( l,\  b\right)}{\partial {b}^2} $$

where *l* is axial position in direction of the gas flow, *b* is vertical position perpendicular to the direction of the gas flow*, v*(*b*) is velocity profile, *C(l,b)* is reactant concentration profile, and *D* the gas-phase diffusion coefficient of reactant species. Gas flow velocity changes from maximum value at the center of reactor tube to 0 value at the surface of wafer (Fig. 3). It increases faster at the center compared to the wafer surface. The gas velocity profile induces the boundary layer thickness ∆_*l*_ in CVD process. Boundary layer thickness is given by [31–33]

2 $$ {\varDelta}_l={\left(\frac{l}{\rho u/\mu}\right)}^{\frac{1}{2}} $$

where *ρ*, *u*, and *μ* are mass density, flow density, and viscosity, and *ρu*/*μ* is called the Reynolds number. For deposition process, reactant gases must diffuse through varying boundary layer thickness ∆_*l*_ shown by Eq. (2) toward downstream to reach the deposition surface (Fig. 3). It shows that the deposition process on wafer surface depends on *S*_*d*_ value. It is well known that reactant species are consumed going downstream in CVD process [31, 34, 35]. Purushothaman and Jeganathan [34] have shown that gallium vapor pressure during growth decreases with increase in *S*_*d*_ value. Menzel et al. [35] have also studied the decrease in metal vapor concentration with *S*_*d*_ for nanowire growth using thermal CVD. Consequently, the concentration of reactant species is depleted with increase in *S*_*d*_ value. Therefore, diameter of Ga_2_O_3_ nanowires is reduced due to increase in ∆_*l*_ and depletion of reactant species with increase in *S*_*d*_.

It is noted that catalyst droplets on tip of nanowires have not been present as shown by circles in inset of Fig. 2b, c. However, clusters were noticed in the root of nanowires. Dotted circle in inset of Fig. 2d shows the cluster (~600 nm) in nanowire root. This indicates the preferential nanowire nucleation follow root growth. Nanowire nucleation within catalyst depends on material-catalyst phase diagram, interfacial parameters, and contact angle of catalyst-nucleus and nucleus-substrate [36]. The noted large size of root cluster is due to supersaturation in catalyst alloy induced by metal vapor pressure at high temperature [37]. Adatom diffusion and direct nucleation of vapors play the crucial role in this root mediated Ga_2_O_3_ nanowires growth. These results show that diameter of root grown Ga_2_O_3_ nanowires can be tuned using *S*_*d*_ and it decreases with increase in *S*_*d*_ value under CVD process.

**Fig. 4:**
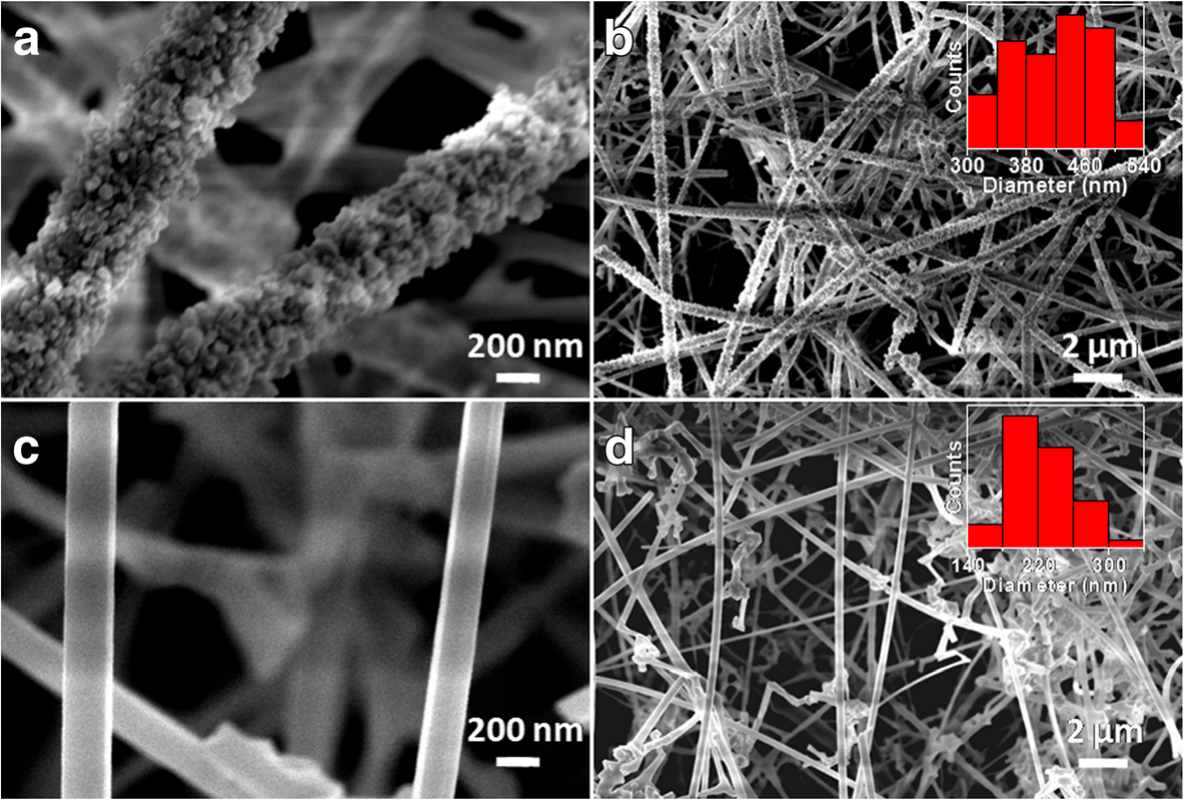
FESEM images of Ga_2_O_3_ nanowires grown with **a**, **b** Ar:O_2_ total flow rate as 160 mL/min (120:40 mL/min) and **c**, **d** Ar/O_2_ flow rate ratio as 1/3(20:60 mL/min) indicating deteriorated nanowire morphology at higher flow rate

Effect of total gas flow rates and mixer ratios of Ar/O_2_ gases on the growth of Ga_2_O_3_ nanowires (900 °C for 30 min with *S*_*d*_ of 4 cm) have been studied. Morphology of nanowires grown with total gas flow rate of 160 mL/min (Ar/O_2_:3/1) is shown in Fig. 4a, b. This is to be compared with sample shown in Fig. 2c, d which was grown with gas flow rate of 80 mL/min (Ar/O_2_:3/1). Surface morphology of nanowires become rough as clustered surface with higher diameter mainly in range of ~300–540 nm (inset of Fig. 4b) at higher total gas flow rate is keeping gas mixer ratio constant. Clusters on nanowire surface grew due to oversupply of source vapor, which can lead to instability in nucleation events [38, 39]. It shows that the morphology of nanowires gets deteriorated with an increase in the total gas flow rates to higher value. Nanowire growth with gas mixer ratio Ar/O_2_:1/3 (80 mL/min) are shown in (Fig. 4c, d). The change in gas mixer ratio does not deteriorate nanowire morphology and exhibit smoother surface with diameter mainly in range of ~140–300 nm (inset Fig. 4d). However, it was observed by XRD that the higher flow rate of O_2_ gas relative to Ar gas induces poor crystalline quality of Ga_2_O_3_ nanowires.

**Fig. 5:**
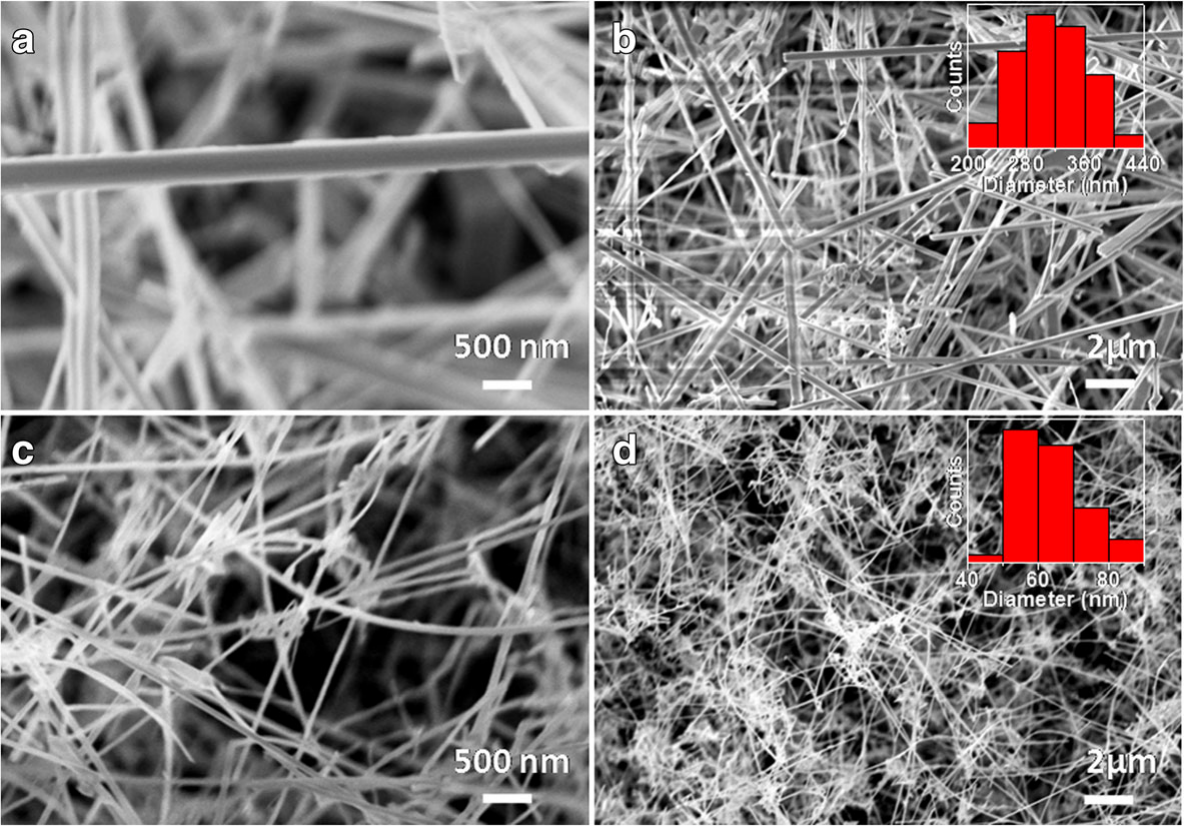
FESEM images of Ga_2_O_3_ nanowires **a**, **b** grown at 850 °C with 50-nm Au nanoparticles and **c**, **d** grown at 800 °C with 50-nm Au nanoparticles. Comparisons of Ga_2_O_3_ nanowires shown by images (**a**, **b**) and (**c**, **d**) indicate the dependence of nanowire diameter on growth temperature

Systematic study of Ga_2_O_3_ nanowires has been carried out at different growth temperatures. There was no growth of nanowires at 1100 °C due to Au nanoparticles desorption from sapphire substrate at high temperature. At 1000 °C, poor growth of nanowires with uncontrolled diameter was observed. However, the morphology and diameter of nanowires are controlled at a growth temperature of 900 °C using *S*_*d*_ (Fig. 2). Morphology of nanowires grown at lower temperature of 850 °C is shown in Fig. 5a, b. Nanowires are straight with uniform diameter along the length. Diameter distribution of these nanowires is shown by histogram (inset of Fig. 5b) indicating diameter range mainly in 240–400 nm. With further reduction in growth temperature to 800 °C (Fig. 5c, d), nanowire diameter was reduced to range mainly in 50–90 nm (inset of Fig. 5d) with nanowires curved in nature. The resulted smaller nanowire diameter is due to the lower adatom mobility and transport of vapor species through boundary layer at lower growth temperature [40]. Comparison of nanowire growth at 850 and 800 °C suggest that Ga_2_O_3_ nanowire diameter is also a function of growth temperature. The diameter of nanowires grown at higher temperature (900, 850 °C) is much larger than the size of catalyst nanoparticles (50 nm). In case of nanowire growth at 800 °C, diameter is close to the size of catalyst nanoparticles. This demonstrates the diameter corelation with catalyst size. Higher vapor pressure of gallium metal at relatively higher growth temperatures induces supersaturation in catalyst alloy which results in larger size catalyst alloy formation [37]. Therefore, nanowire diameter does not depend on the size of catalyst nanoparticles at higher growth temperature. Using growth temperature in this case, nanowire diameter can be tuned from few hundred nanometers to several tens of nanometers. However, poor crystalline quality was observed at lower growth temperature (800 °C). Further, nanowire growths were not observed at lower temperature ≤700 °C due to unfulfilled growth conditions for catalyst assisted growth process as employed in the present work.

**Fig. 6:**
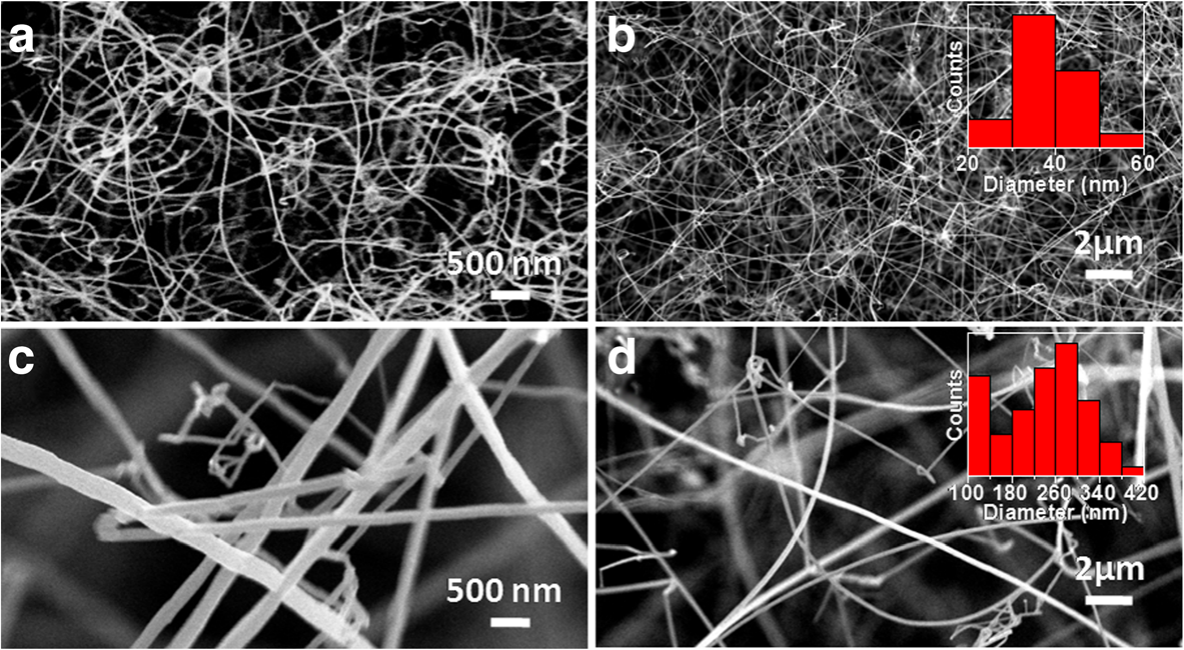
FESEM images of Ga_2_O_3_ nanowires **a**, **b** grown at 800 °C with 20-nm Au nanoparticles and **c**, **d** grown at 850 °C with 20-nm Au nanoparticles. Comparisons of Ga_2_O_3_ nanowires shown by images (**a**, **b**) and (**c**, **d**) indicate the dependence of nanowire diameter on growth temperature

**Fig. 7:**
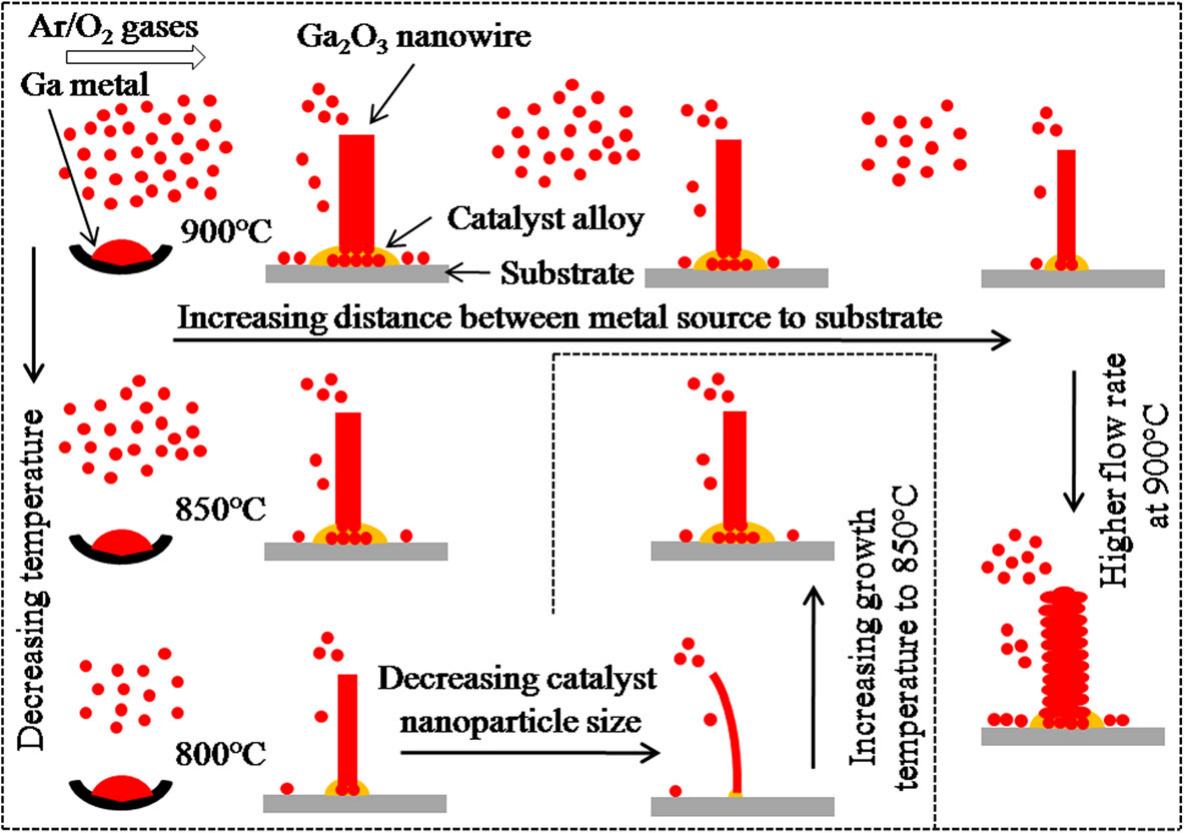
Growth schematic for diameter tuning of Ga_2_O_3_ nanowires at various growth conditions in CVD system. Root growths of Ga_2_O_3_ nanowires where processes of surface diffusion transport and direct nucleation of species from vapor phase dominate for the root growth have been demonstrated. Diameter tuning of Ga_2_O_3_ nanowires have been illustrated by metal source to substrate separation distances on the basis of reactant vapor species and boundary layer in CVD process. Effects of gas flow rate on nanowire morphology are shown. Growth temperatures and catalyst nanoparticle size are demonstrated where temperature-dependent vapor pressure and adatom mobility play crucial role to control the nanowire diameter

Ga_2_O_3_ nanowires growth at 800 °C using smaller Au nanoparticles of 20 nm size (gas flow rates of 80 mL/min: Ar/O_2_:3/1, *S*_*d*_ 4 cm) results the nanowire diameter down to few tens of nanometer as shown in Fig. 6a, b with diameter histogram as an inset. As-grown nanowires are entangled and curved in nature. It shows that nanowire diameter below 50 nm can be tuned using lower growth temperature, such as 800 °C and smaller (20 nm) Au nanoparticles as catalyst. With increase in growth temperature to 850 °C, most of nanowire diameter become much larger as shown in Fig. 6c, d with diameter histogram as an inset. This further confirms the independence of nanowire diameter from size of catalyst nanoparticles at growth temperature of 850 °C. Growth schematic for diameter tuning of root grown Ga_2_O_3_ nanowires has been schematically illustrated in Fig. 7. Various experimental conditions such as separation distance between metal source and substrate, growth temperature, gases flow rates, and catalyst nanoparticle sizes are shown. In root grown Ga_2_O_3_ nanowires, surface diffusion transport and direct nucleation of species from vapor phase dominate for nanowire growth. Top panel of schematic shows that the nanowire diameter decreases with increase in separation distance between metal source and substrate as reactant vapor species depleted downstream and boundary layer thickness increases. Nanowire diameter depends on growth temperature. This is demonstrated in the left side of schematic by various growth temperatures where temperature dependent vapor pressure and adatom mobility play a crucial role to control the nanowire diameter. Low growth temperature and smaller size of catalyst nanoparticle can be used to grow nanowire with smaller diameter as illustrated in the bottom of schematic. The higher flow rate of gases induces the larger nanowire diameter whereas its surface morphology gets deteriorated as shown in the right side of schematic. Thus, diameter of Ga_2_O_3_ nanowires can be tuned from several hundreds of nanometers to few tens of nanometers by exploiting growth conditions in CVD reactor. Different diameters as D1, D2, and D3 of Ga_2_O_3_ nanowires grown at 900 °C were chosen for further comparative investigations.

**Fig. 8:**
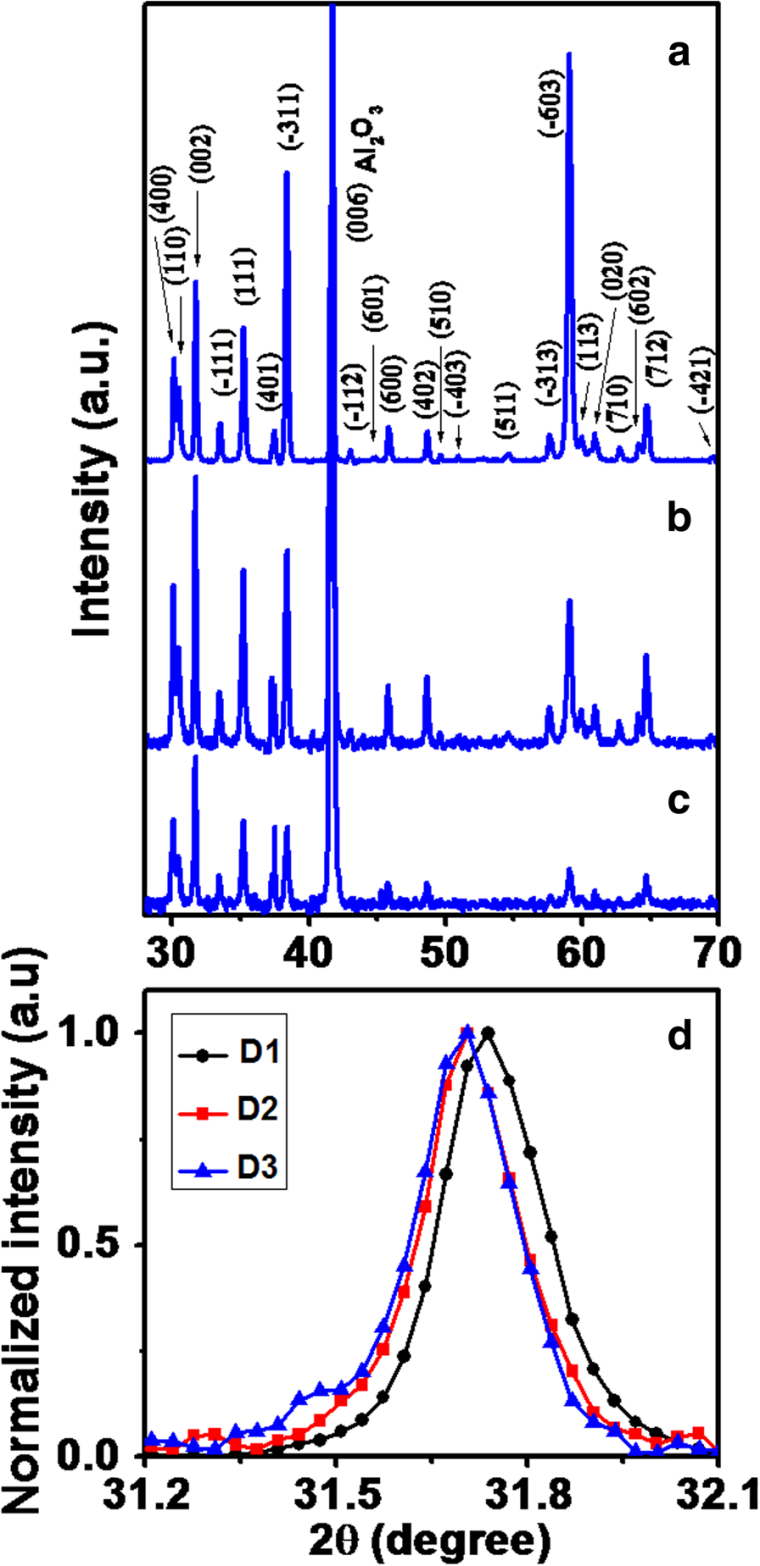
XRD patterns of Ga_2_O_3_ nanowires grown at source to substrate distances of **a** 2 cm (D1), **b** 4 cm (D2), and **c** 6 cm (D3) where Miller indices (*hkl*) of peaks according to JCPDS confirms the monoclinic crystal structure of beta (*β)* phase of Ga_2_O_3_ nanowires. **d** Comparative XRD peak corresponding to (002) plane of Ga_2_O_3_ nanowires indicating change of strain with nanowire diameter

Structural investigations of the Ga_2_O_3_ nanowires with different diameters have been carried out using XRD patterns as shown in Fig. 8 (a), (b), and (c) for the cases D1 (nanowire diameters 500–700 nm), D2 (nanowire diameters 300–400 nm), and D3 (nanowire diameters 150–250 nm), respectively. XRD patterns from ensembles of nanowires grown in random direction consist of number of closely spaced sharp diffraction peaks. The absence of any impurity-related XRD peaks substantiates the high purity of Ga_2_O_3_ nanowires. These peaks are marked as Miller indices (*hkl*) of *β*-Ga_2_O_3_ (JCPDS file number 43–11012) indicating monoclinic crystal structure of as-grown nanowires. Sharp XRD signals indicate high crystalline quality of as-grown *β*-Ga_2_O_3_ nanowires. Intensity of XRD patterns decreases with decrease in nanowire diameters. In case of D1, $\beta$-Ga_2_O_3_ nanowires show dominant $\left(\overline{6}03\right)$ plane which decreases in intensity in D2 and further in D3. $\beta$-Ga_2_O_3_ nanowires exhibit the dominant (002) plane in case of D2 and D3. Zoom XRD spectra corresponding to (002) plane of $\beta$-Ga_2_O_3_ nanowires for D1, D2, and D3 cases have been shown in Fig. 8d. The shift in position of (002) plane peaks corresponding to small increase in inter-planer spacing for D1 to D3 represents the change in structural strain with nanowire diameter. Estimated change in structural strain from D1 to D2 was ~0.1% and from D1 to D3 was ~0.14%. Since XRD investigation has been performed on ensembles of β-Ga2O3 nanowires which were grown randomly on a substrate in different directions, it is difficult to correlate the plane orientation with respect to nanowire diameter in the present work.

**Fig. 9:**
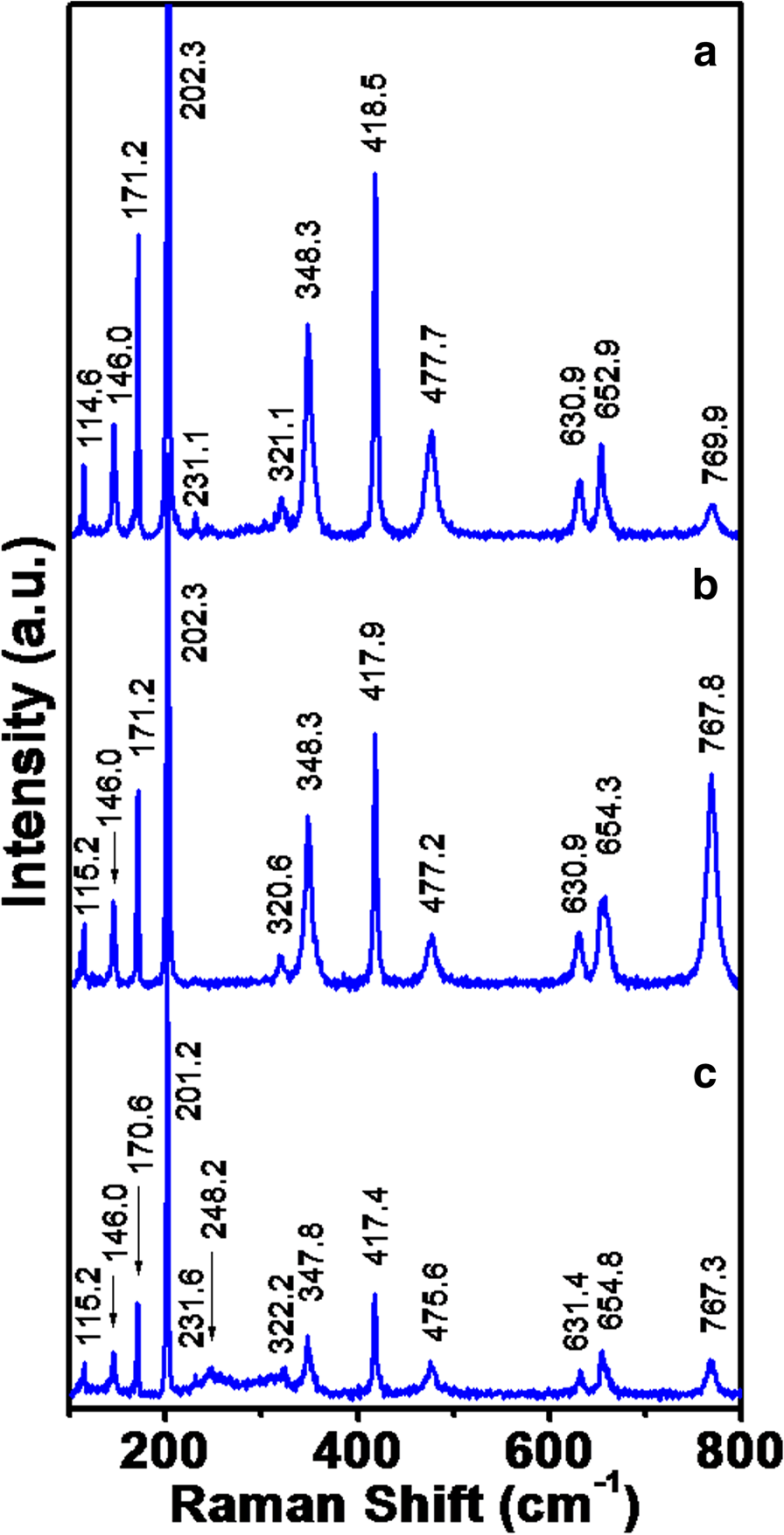
Raman spectra of Ga_2_O_3_ nanowires grown at source to substrate distances of **a** 2 cm (D1), **b** 4 cm (D2), and **c** 6 cm (D3) where the phonon modes correspond to beta (*β)* phase of Ga_2_O_3_

To study various phonon modes in $\beta$-Ga_2_O_3_ nanowires, Raman spectra have been measured using 514 Ar ion laser. Raman spectra of $\beta$-Ga_2_O_3_ nanowires for the cases D1, D2, and D3 are shown in Fig. 9 (a), (b), and (c), respectively. The *β*-Ga_2_O_3_ having monoclinic crystal structure exhibits 27 optical modes at Г-point (Brillouin zone center) with irreducible representation [41]

$$ {\varGamma}^{opt}=10{A}_g\kern0.5em  + 5{B}_g\kern0.5em +4{A}_u\kern0.5em +8{B}_u $$

Modes with *A*_*u*_ and *B*_*u*_ symmetry are infrared active and modes with *A*_*g*_ and *B*_*g*_ symmetry are Raman active. Raman modes under non-resonant conditions depend on crystal orientation and polarization configurations specified by selection rules [41, 42]. On the basis of Raman study, unit cell of β-Ga_2_O_3_ consists of two formula unit cells as octahedral (Ga_2_O_6_) and tetrahedral (GaO_4_) [43, 44]. Low energy phonon modes (100–300 cm^−1^) correspond to the liberation and translation of tetrahedra-octahedra chains, moderate energy phonon modes (300–500 cm^−1^) correspond to deformation of Ga_2_O_6_ octahedra, and high energy phonon modes (600–800 cm^−1^) correspond to stretching and bending of GaO_4_ tetrahedra. In our previous report, Raman measurements from *β*-Ga_2_O_3_ nanowires and bulk single crystal have been reported [45]. In the present case, the Raman peaks are in general agreement with the peaks reported for nanowires in [45], but we have observed further small shift as the nanowire diameter changes. For example, phonon mode at position 114.6 cm−1 for D1 shifts to the position 115.2 cm−1 for D2 and D3, while strongest phonon mode at position 202.3 cm−1 for D1 and D2 shifts to the position 201.2 cm−1 for D3. Similarly, phonon mode at position 652.9 cm−1 for D1 shifts to the position 654.3 cm−1 for D2 and 654.8 cm−1 for D3. While phonon mode at position 769.9 cm−1 for D1 shifts to the position 767.3 cm−1 for D3. It is reported that blue shift in the phonon modes is due to internal strain in the nanowires and red shift is due to the presence of defects in nanowires, such as O vacancies which cause abnormality in the Ga–O bond vibration [44, 46]. These small-shifted Raman positions in different nanowire diameters indicate variation in strain level as calculated from XRD measurements besides defects.

**Fig. 10:**
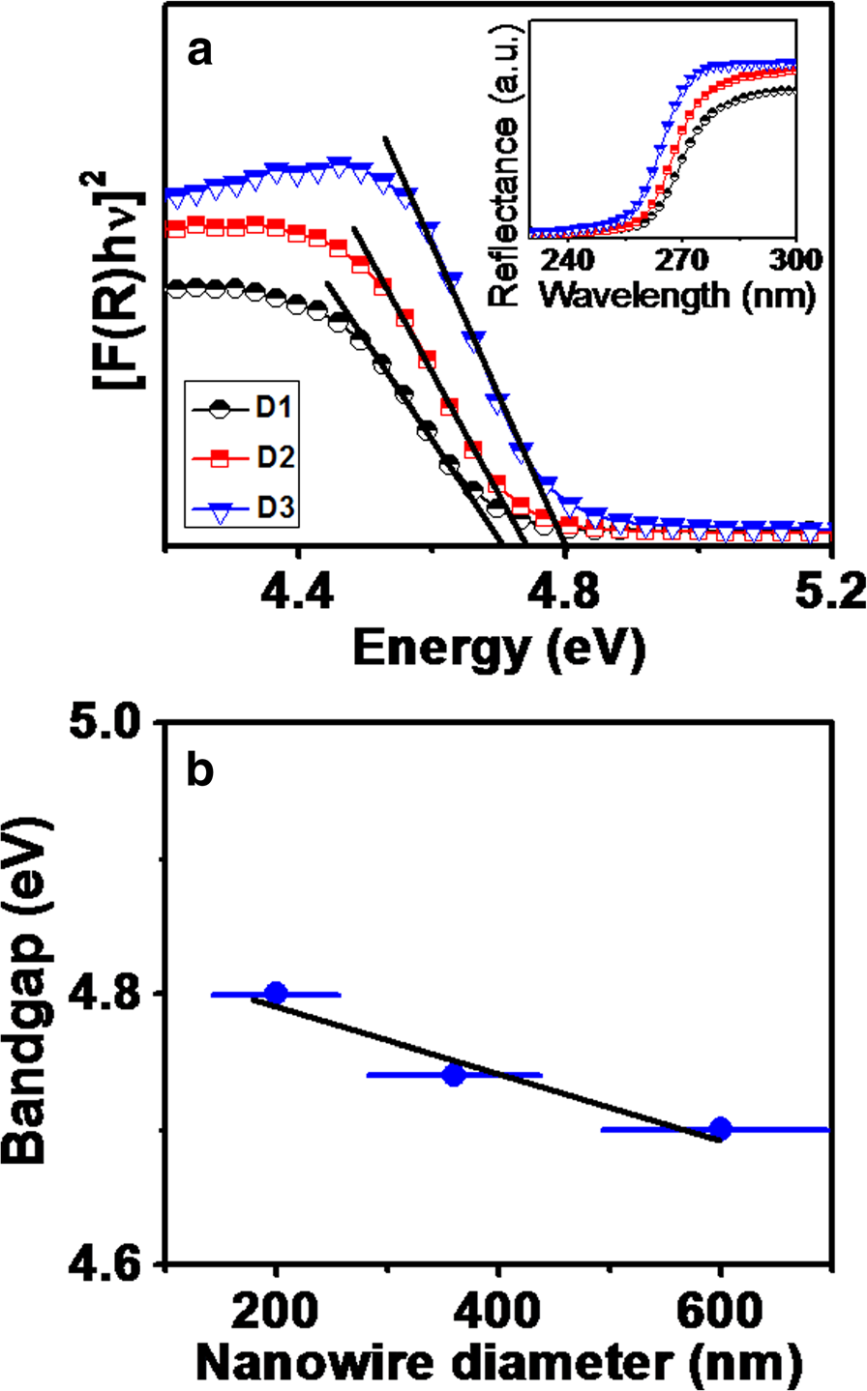
**a** A plot between [F(R) hν]^2^ versus photon energy (hν) for $\beta$-Ga_2_O_3_ nanowires with decreasing diameters from D1 to D3 where band-gap (E_g_) were estimated by translating respective reflectance curve (shown in inset) to Kubelka-Munk (K-M) function F(R). The increase in band-gap of $\beta$-Ga_2_O_3_ nanowires with its diameter is shown in (**b**)

The band-gap of $\beta$-Ga_2_O_3_ nanowires have been determined using UV-vis spectrophotometer. The plot between [F(R)hν]^2^ versus photon energy (hν) for different diameters of $\beta$-Ga_2_O_3_ nanowires as D1, D2, and D3 are shown in Fig. 10a with respective measured reflactance curves in its inset. Band-gap (E_g_) was estimated by translating reflectance curve from UV-vis spectrophotometer to Kubelka-Munk (K-M) function F(R) and then correlating E_g_ with K-M function F(R) by well known equation [47] as

3 $$ {\left[\mathrm{F}\left(\mathrm{R}\right) h\nu \right]}^2 = B\left( h\nu -{E}_g\right) $$

where *B* is a constant. Besides the large number of investigations on $\beta$-Ga_2_O_3_, there are still disputes in regard of its band-gap value and it has been reported within the range of 4.7–4.9 eV [11, 16, 48, 49]. Estimated band-gap of the nanowires as D1, D2, and D3 cases comes out to be 4.70, 4.74, and 4.80 eV. It shows that band-gap of nanowires depends on the diameter and it increases from 4.70 to 4.80 eV with decrease in diameter from several hundreds of nanometers to few hundreds of nanometers. Band-gap varies approximately linearly with decrease in nanowire diameters (Fig. 10b). Size dependent band-gap variations have been studied in strained ZnO nanowires having diameters in the range 100–1000 nm [30]. The band-gap value can be altered due to surface reconstruction (increased surface to volume ratio) and surface strain in different sizes of nanowire [30, 50]. As indicated by Raman spectra, nanowires of different diameters undergo distinct variation of phonon modes related to octahedral (Ga_2_O_6_) and tetrahedral (GaO_4_) unit cell of β-Ga_2_O_3_. Therefore, axial bond length variation sensitive to core and radial bond length variation sensitive to surface of strained nanowire of different diameters [30, 50] can cause the shifting of band-gap values. However, surface adsorption and crystal defects and impurities can also affect the band-gap value. Therefore, detected diameter-dependent band-gap variation of β-Ga_2_O_3_ nanowires need to be further investigated in details for physical mechanisms.

## Conclusions

In conclusions, growth of $\beta$-Ga_2_O_3_ nanowires with different diameters has been explored under various experimental conditions using CVD technique. Diameter of $\beta$-Ga_2_O_3_ nanowire grown by catalyst alloy-mediated root growth can be tuned by adjusting growth conditions. The separation distance between metal source and substrate controls the nanowire diameter on the basis of boundary layer thickness and reactant vapor species. Nanowire diameter decreases with increase in the separation distance. Higher gas flow rate induces larger diameter; however, nanowire morphology gets deteriorated. Diameter of nanowires depends on growth temperature and catalyst nanoparticles size. Diameter of nanowire grown at higher temperature (850–900 °C) does not depend on catalyst nanoparticle size. The smaller diameter of nanowire (<50 nm) can be obtained using lower growth temperature (800 °C) and smaller catalyst nanoparticles size (20 nm). Ga_2_O_3_ nanowires exhibit $\beta$-phase with monoclinic crystal structure. XRD and Raman measurements indicate the changes in structural strain with nanowire diameter. Band-gap of these nanowires depends on its diameter, and it increases from 4.70 to 4.80 eV with decrease in nanowire diameter from several hundreds of nanometers to few hundreds of nanometers.
